# The efficacy and safety of disitamab vedotin plus immunotherapy in locally advanced or metastatic solid tumors: a systematic review and meta-analysis

**DOI:** 10.3389/fimmu.2026.1763542

**Published:** 2026-02-25

**Authors:** Jianjun Ye, Zeyu Chen, Jie Feng, Xinyang Liao, Shiyu Zhang, Qihao Wang, Lei Zheng, Tiancheng Liu, Qiang Wei, Yige Bao

**Affiliations:** 1 Department of Urology and Institute of Urology, West China Hospital, Sichuan University, Chengdu, China; 2 West China School of Medicine, Sichuan University, Chengdu, China; 3 State Key Laboratory of Oral Diseases & National Clinical Research Center for Oral Diseases, Department of Orthodontics, West China Hospital of Stomatology, Sichuan University, Chengdu, China

**Keywords:** disitamab vedotin, HER2, immunotherapy, meta-analysis, solid tumors

## Abstract

**Background:**

The combination of disitamab vedotin (DV), a novel human epidermal growth factor receptor 2 (HER2)-targeting antibody-drug conjugate, with immunotherapy represents a promising strategy for locally advanced or metastatic solid tumors. However, comprehensive evidence regarding its efficacy and safety is lacking. This systematic review and meta-analysis aimed to synthesize available data on this combination regimen.

**Methods:**

We systematically searched PubMed, Scopus, Embase, and the Cochrane Library for studies published up to December 31, 2025. The primary outcomes were objective response rate (ORR) and treatment-related adverse events (TRAEs). Secondary outcomes included disease control rate (DCR) and median progression-free survival (mPFS). Pooled analyses were performed using a random-effects model.

**Results:**

21 studies involving 1183 patients were included. The pooled ORR was 53% (95% CI: 46%–60%), and the DCR was 82% (95% CI: 77%–86%). The pooled mPFS was 7.8 months (95% CI: 6.6–8.9). Subgroup analyses indicated superior efficacy in urothelial carcinoma, HER2-positive tumors, and first-line treatment settings. Any-grade and grade ≥3 TRAEs occurred in 91.1% and 36.8% of patients, respectively, with a toxicity profile dominated by DV-related adverse events such as fatigue, peripheral neuropathy, and hematological toxicities.

**Conclusion:**

The combination of DV and immunotherapy demonstrates encouraging antitumor activity and a manageable safety profile in patients with locally advanced or metastatic solid tumors, particularly in HER2-expressing populations and when used in the first-line setting. These findings support further investigation of this combination in randomized controlled trials.

**Systematic Review Registration:**

https://www.crd.york.ac.uk/prospero/, identifier CRD420251154446.

## 1 Introduction

The management of locally advanced or metastatic solid tumors remains a formidable challenge in clinical oncology (1). While conventional chemotherapy continues to be a cornerstone, the advent of targeted therapies and immunotherapy, particularly immune checkpoint inhibitors (ICIs), has revolutionized treatment paradigms. ICIs, by reactivating the host immune system against cancer cells, have demonstrated remarkable and durable responses across a spectrum of malignancies (2–4). However, the benefits of immunotherapy are not universal; a significant proportion of patients exhibit primary resistance, and many who initially respond eventually develop acquired resistance (5, 6). This stark reality underscores the critical need for novel combination strategies designed to amplify anti-tumor immunity and overcome the limitations of monotherapy.

Disitamab vedotin (DV) is an innovative antibody-drug conjugate (ADC) that targets the Human Epidermal Growth Factor Receptor 2 (HER2) (7, 8). Its unique design incorporates a hertuzumab-derived antibody with a potent microtubule-disrupting agent, monomethyl auristatin E (MMAE) (8, 9). A key advantage of DV is its ability to exert cytotoxic effects not only in tumors with high HER2 expression but also in those with lower HER2 expression levels, a population often ineligible for traditional HER2-targeted treatments (10, 11). Beyond its direct cytotoxic payload delivery, emerging preclinical evidence suggests that DV can induce immunogenic cell death, a process that may enhance tumor immunogenicity by promoting antigen presentation and fostering a favorable tumor microenvironment (12, 13). This mechanism provides a strong scientific rationale for combining DV with ICIs, with the potential to synergistically boost T-cell-mediated anti-tumor activity and reverse immunotherapy resistance.

Consequently, several clinical trials have begun to explore the combination of DV and ICIs, reporting promising preliminary activity in various solid tumors, such as urothelial carcinoma and gastric cancer (14, 15). However, the current evidence is fragmented across early-phase studies with limited sample sizes. The overall efficacy, safety profile, and consistency of this combination regimen have not been comprehensively evaluated. Therefore, we will conduct this systematic review and meta-analysis to synthesize all available clinical evidence about the efficacy and safety of DV plus immunotherapy, thereby offering crucial insights for clinical decision-making and the design of future definitive trials.

## 2 Methods

The protocol for this systematic review was prospectively registered with the International Prospective Register of Systematic Reviews (PROSPERO; registration number: CRD420251154446). The conduct and reporting of this study adhere to the Preferred Reporting Items for Systematic Reviews and Meta-Analyses (PRISMA) guidelines (16). A completed PRISMA checklist is provided in **Supplementary Table 1** to ensure comprehensive and transparent reporting.

### 2.1 Search selection

A systematic literature search was performed using PubMed, Scopus, Embase, and the Cochrane Library for studies published up to December 30, 2025, with no restrictions on language. The search strategy incorporated the terms “RC48,” “DV,” and “Disitamab vedotin,” combined with the Boolean operator “OR”.

Study selection followed the Population, Intervention, Comparison, Outcomes and Study (PICOS) framework (17), wherein the population of interest consisted of patients diagnosed with locally advanced or metastatic solid tumors; the intervention comprised DV combined with immunotherapy; no comparator was specified; outcomes of interest included objective response rate (ORR), disease control rate (DCR), median progression-free survival (mPFS), and treatment-related adverse events (TRAEs); and no limitations were placed on study designs at the initial screening phase.

Exclusion criteria involved studies with insufficient outcome information, non-original publications such as reviews, editorials, case reports, and letters, as well as unpublished articles and duplicate reports or overlapping cohorts.

### 2.2 Data extraction and endpoints of interest

Two independent reviewers (JJY and ZYC) performed the data extraction, with any discrepancies resolved through consensus. The extracted information encompassed the following: first author’s name, publication year, journal name, country of origin, study type, study period, tumor type, clinical setting, sample size, HER2 expression status, PD-L1 status, DV treatment regimen, immunotherapy regimen, treatment line, primary endpoints, ORR and DCR cases, mPFS, and TRAEs. The primary outcomes of interest were ORR and TRAEs, while secondary outcomes included DCR and mPFS. Only studies that reported, or from which it was possible to derive, at least one of the primary outcomes were considered eligible for inclusion.

### 2.3 Quality assessment

The standard risk of bias in nonrandomized studies of interventions version I tool (ROBINS-I) was used to evaluate the risk of bias of the included studies (18). Each of the seven specific domains—bias due to confounding, bias due to selection of participants, bias in classification of interventions, bias due to deviations from intended interventions, bias due to missing data, bias in measurement of outcomes and bias in selection of the reported result—was evaluated as serious, moderate, or low. The assessment was conducted independently by two authors (JJY and ZYC), with any discrepancies resolved through discussion.

### 2.4 Data consolidation

This study conducted a single-arm meta-analysis of proportions to systematically evaluate the efficacy of DV combined with immunotherapy in patients with locally advanced or metastatic solid tumors. All statistical analyses were carried out using R software (version 4.1.3) with the “metafor”, “meta”, “dmetar”, and “ggplot2” packages.

For ORR and DCR, pooled proportions and 95% confidence intervals (CIs) were calculated using the DerSimonian and Laird random-effects model (19). Proportions falling outside the 0.2–0.8 range were transformed via the logit method, and double-arcsine transformation was applied for studies with zero events. Survival outcome (mPFS), was synthesized based on reported median values and 95% CIs. When complete data were unavailable, Kaplan–Meier curves were digitized using Engauge Digitizer (version 11.3) to estimate median times and corresponding 95% CIs; studies without reported median values or CI bounds were excluded. Safety outcomes were summarized as counts and percentages with 95% CIs.

Heterogeneity was evaluated with the I² statistic, considering values exceeding 30% with a p-value below 0.10 as indicative of significant heterogeneity. Publication bias was assessed using funnel plots and Egger’s linear regression test. Where applicable, exploratory subgroup analyses—such as by tumor type, HER2 expression level and treatment line—were conducted to explore potential sources of heterogeneity and identify patient subgroups that may derive greater benefit.

## 3 Result

### 3.1 Search results and basic characteristic

A total of 396 records were initially identified through the literature search, of which 21 studies met the eligibility criteria and were included in the analysis (14, 15, 20–38) (**Supplementary Figure 1**). The key characteristics of these studies are summarized in **Table 1**.

**Table 1:** Main characteristics of the studies included in the meta-analysis.

First author	Year	Journal	Country	Study type	Study period	Tumor type	Clinical setting	Sample size	HER2 expression	PDL1 status	DV treatment regimen	ICIs treatment regimen	Treatment line	Primary endpoints
Sheng (33)	2025	N Engl J Med	China	Phase III trial (RC48-C016 trial)	2022.6-2025.3	Urothelial carcinoma	Unresectable; locally advanced or metastatic; Without systemic chemotherapy history	243	HER2 3+: 61 HER2 2+: 127 HER2 1+: 55	Combined positive score <1: 68/125; Combined positive score ≥1: 57/125	2.0 mg/kg, q2w	Toripalimab (3.0 mg/kg), q2w	First-line	Progression- free survival; overall survival
Lin (34)	2025	Ther Adv Med Oncol	China	Multicenter, retrospective study	2021.6-2024.1	Urothelial carcinoma	Metastatic	63	HER2 3+: 15 HER2 2+: 25 HER2 1+: 13 HER2 0+: 10	≥10%: 9 <10%: 42 Unknown: 12	2.0 mg/kg, q2W	Toripalimab (3mg/kg) or Tislelizumab (200 mg), q3W	First-line	Efficacy and safety
Zhou (35)	2025	BMC Urology	China	Single-center, retrospective study	2022.8-2024.6	Urothelial carcinoma	Locally advanced or metastatic	71	HER2 3+: 14 HER2 2+: 37 HER2 1+: 5 HER2 0: 2 HER2-U: 13	Na	2.0 mg/kg, q2W	Toripalimab (240 mg) or Tislelizumab (200 mg), q3W	No restrictions	ORR
Liu (36)	2025	BMC Cancer	China	Multicenter, retrospective study	2021.4-2024.3	Urothelial carcinoma	Metastatic	30	HER2 3+: 4 HER2 2+: 10 HER2 1+: 6 HER2-U: 10	Na	Na	Na	No restrictions	Progression- free survival; overall survival
Qu (37)	2025	Urol Oncol	China	Retrospective study	2017.12-2024-12	Urothelial carcinoma	Locally advanced or metastatic	108	HER2 3+: 9 HER2 2+: 49 HER2 1+: 30 HER2 0: 16 HER2-U: 5	Negative: 57 Positive: 22 Unknown: 29	Na	Na	No restrictions	Overall survival
Wang D (22)	2025	BMC Cancer	China	Multicenter, retrospective study	2021.12-2024.6	Urothelial carcinoma	Locally advanced or metastatic; Unresectable; HER2-N	20	HER2 1+: 17 HER2 0: 6	Na	2.0 mg/kg, q2W	Determined by physician	No restrictions	ORR
Yan (38)	2025	Ann Med	China	Multicenter, retrospective study	2021.6-2023.9	Gastric cancer	Pre-treated HER2 overexpressed advanced gastric cancer	36	HER2 3+: 13 HER2 2+: 23	CPS<1: 5 CPS ≥1: 21 Unknown: 10	2.5 mg/kg, q2-3W	tislelizumab, toripalimab, sintilimab, nivolumab or penpulimab	Non-first line	Efficacy and safety
Yao (21)	2025	World J Urol	China	Multicenter, retrospective study	2022.1- 2024.7	Urothelial carcinoma	Locally advanced or metastatic; Prior platinum-based chemotherapy history	51	HER2 3+: 7 HER2 2+: 31 HER2 1+: 10 HER2 0+: 3	Na	2.0 mg/kg, q2W	Tislelizumab (200 mg) or Toripalimab (3 mg/kg), q3W	Non-first line	ORR; DCR
Zhang (20)	2025	Discov Oncol	China	Single-center, retrospective study	2023.1-2023.12	Urothelial carcinoma	Locally advanced or metastatic; HER2 ≥ 1+	27	HER2-P: 17 HER2 1+: 10	PDL1 ≥ 1:16 PDL1 < 1:10	2.0 mg/kg, q2W	Tislelizumab (200 mg) or Toripalimab (3 mg/kg), q3W	First line	Efficacy and safety
Zhou L (14)	2025	Ann Oncol	China	Phase Ib/II study (RC48-C014 trial)	2020.8-2021.12	Urothelial carcinoma	Untreated or chemo-refractory; Locally advanced or metastatic	41	HER2 3+: 5 HER2 2+: 19 HER2 1+: 14 HER2 0: 3	PDL1-high: 16 PDL1-low: 25	An escalating dose of 1.5 or 2.0 mg/kg, q2W	Toripalimab: 3.0 mg/kg, q2W	No restrictions	Tolerability and safety
Ng (23)	2025	Cancer Immunol Immunother	China	CUDA-UTU CCG, Multicenter, retrospective study	2021.6-2023.12	Upper tract Urothelial carcinoma	Metastatic	198	HER2-P: 144 HER2-N: 40 HER2-U:14	PDL1 (≥ 1% or CPS ≥ 10): 72 PDL1 (< 1% or CPS < 10): 82	Determined by physician, q3W	Determined by physician, q3W	No restrictions	ORR
Ge (24)	2025	J Transl Med	China	Multicenter, retrospective study	2022.10-2024.5	Urothelial carcinoma	Locally advanced or metastatic	25	Na	Na	2.0 mg/kg, q2W	Toripalimab (3 mg/kg) or Tislelizumab (3 mg/kg), q3W	No restrictions	Efficacy and safety
Dong (29)	2025	Sci Rep	China	Multicenter, retrospective study	2021.7-2023.4	Gastric cancer	Advanced; Failure of second-line or subsequent treatments	34	HER2 3+: 10 HER2 2+: 24	PDL1 ≥ 1:22 PDL1 < 1:12	2.5 mg/kg, q2W	Determined by physician	Non-first line	Efficacy and safety
Wang Y	2025	EClinical-Medicine	China	Phase I trial (NCT0428 0341)	2020.7-2022.8	Solid tumors	Locally advanced unresectable or metastatic gastric cancer or other advanced solid malignant tumors	56	HER2 3+: 16 HER2 2+: 30 HER2 1+: 10	PDL1 ≥ 1:12 PDL1 < 1:16 PDL1-U: 28	2.0 or 2.5 mg/kg, q2W	Toripalimab: 3.0 mg/kg, q2W	No restrictions	Safety and tolerability
Chen J (26)	2024	Clin Genitourin Cancer	China	Multicenter, retrospective study (5)	2021.7-2023.8	Urothelial carcinoma	Metastatic	73	Na	Na	2.0 mg/kg, q2W	Determined by physician	No restrictions	Efficacy and safety
Zhu (25)	2024	Front Pharmacol	China	Single-center, retrospective study	2010-2022	Urothelial carcinoma	Locally advanced or metastatic; Failed first-line treatment	16	HER2 2+: 10 HER2 1+: 6	Na	2.0 mg/kg, q2W	Tislelizumab: 200 mg, q3W	Non-first line	Efficacy and safety
Chen M (28)	2023	Cancer Immunol Immunother	China	Multicenter, retrospective study	2021.7-2022.4	Urothelial carcinoma	Locally advanced or metastatic	18	Na	Na	Na	Determined by physician	No restrictions	Efficacy and safety
Xu (27)	2023	Cancer Med	China	Multicenter, retrospective study	2021.8-2022.10	Urothelial carcinoma	Locally advanced or metastatic	30	HER2-P: 9 HER2-N: 3 HER2-U:18	Na	2.0 mg/kg, q2W	Tislelizumab (200 mg) or Toripalimab (3 mg/kg), q3W	No restrictions	Efficacy and safety
Zhou Y (30)	2023	Aging (Albany NY)	China	Single-center, retrospective study	2019.7-2023.6	Solid tumors	Advanced or metastatic	34	HER2 3+: 5 HER2 2+: 14 HER2 1+: 5 HER2 0: 3 HER2-U: 7	Na	120mg q2W: 28 <120mg q2W: 6	Determined by physician	No restrictions	Efficacy and safety
Wang P (31)	2023	BMC Cancer	China	Single-center, retrospective study	2021.7-2022.12	Solid tumors	Locally advanced or metastatic; HER2 ≥ 1+; Failure of systemic chemotherapy	13	Na	Na	2.5 mg/kg, q2W	Na	Non-first line	DCR
Nie (32)	2023	BMC Cancer	China	Multicenter, retrospective study	2021.8-2022.1	Gastric cancer	Low advanced or metastatic; Failed from two or more lines of prior therapy	25	HER2-P: 17 HER2-low: 8	Na	2.5 mg/kg, q2W	Tislelizumab: 200 mg, q3W	Third-line	Efficacy and safety

DV, Disitamab vedotin; ICIs, immune checkpoint inhibitors; HER2-P, HER2-positive; HER2-N, HER2-negative; HER2-U, HER2-unknown; Na, Not available; ORR, Objective response rate; DCR, disease control rate.

The included studies encompassed 1183 patients with locally advanced or metastatic solid tumors who were treated with DV in combination with immunotherapy. In terms of study design, only three studies (14.3%) were prospective clinical trials (14, 15, 33), five (23.8%) were single-center retrospective studies (20, 25, 30, 31, 35), and the majority (61.9%) were multicenter retrospective analyses (21–24, 26–29, 32, 34, 36–38). With regard to tumor types, urothelial carcinoma (UC) was the most frequently represented (n = 15, 71.4%), followed by gastric cancer (GC) (n = 3, 14.3%) and other solid tumors (n = 3, 14.3%). Regarding treatment lines, the combination of DV and immunotherapy was used as first-line treatment in three study (14.3%), as non-first-line therapy in six studies (28.6%), while the remaining studies (57.1%) did not specify the treatment line.

### 3.2 Primary endpoints: ORR

**Figure 1:**
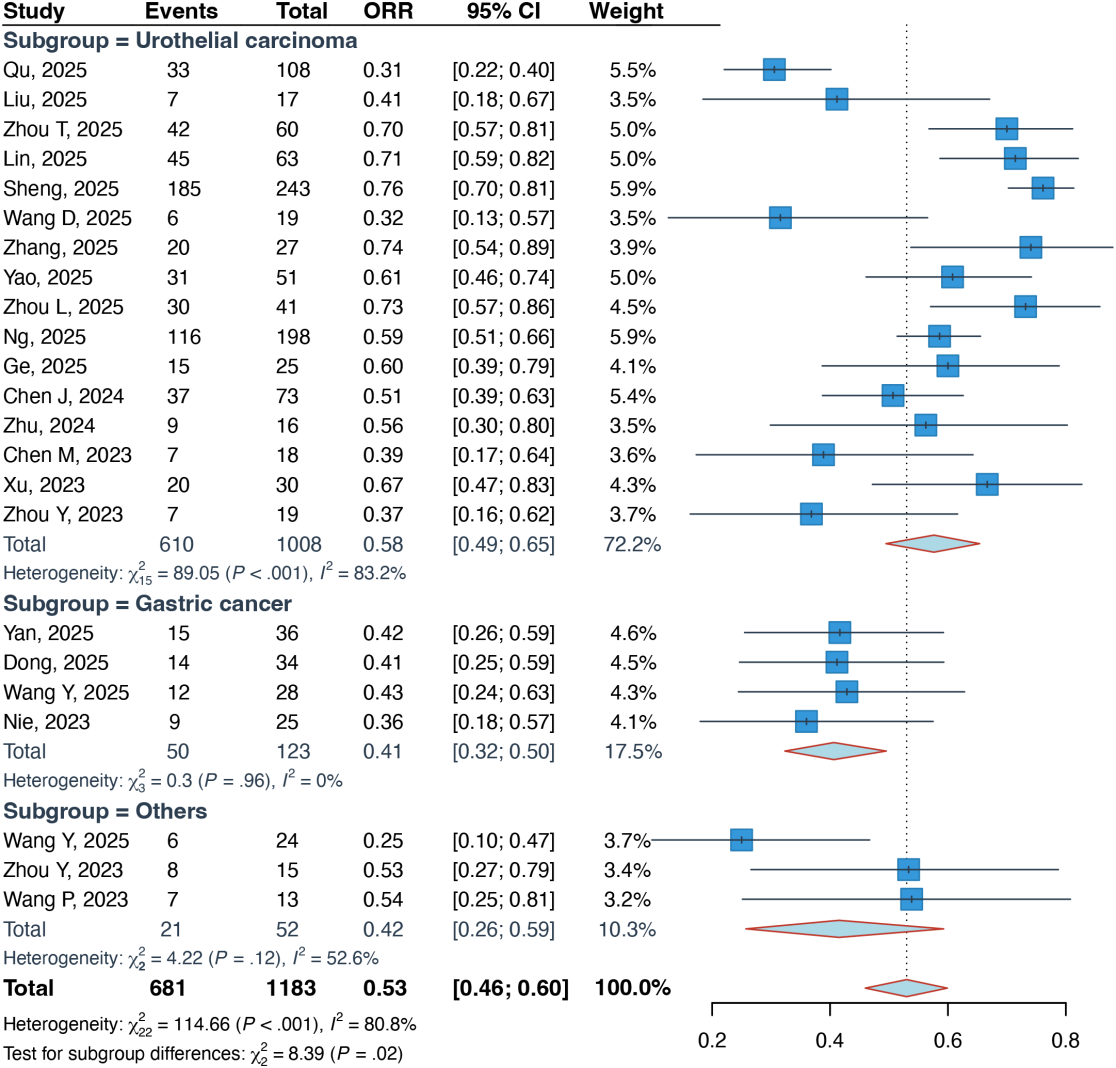
Forest plots about the pooled ORR in patients with locally advanced or metastatic solid tumors. ORR, objective response rate.

A total of 21 studies comprising 1183 evaluable patients were included in the pooled analysis of ORR. The overall ORR was 53% (95% CI: 46%–60%), with significant heterogeneity observed across studies (I² = 80.8%, p < 0.01) (**Figure 1**). Subgroup analyses revealed variations in efficacy based on tumor type, HER2 expression, and treatment line. Patients with UC achieved a higher ORR of 58% (95% CI: 49%–65%), compared to 41% (95% CI: 32%–50%) in those with GC (P = 0.02) (**Figure 1**). Tumors with HER2-positive expression exhibited a significantly better response, with an ORR of 67% (95% CI: 56%–77%), versus 50% (95% CI: 41%–60%) in HER2-negative cases (P = 0.02) (**Supplementary Figure 2**). Furthermore, the combination therapy demonstrated superior efficacy when used in the first-line setting (ORR: 71%; 95% CI: 65%–76%) compared to non-first-line therapy (ORR: 52%; 95% CI: 46%–59%) (P < 0.01) (**Supplementary Figure 3**).

### 3.3 Primary endpoints: most prevalent TRAEs

**Figure 2:**
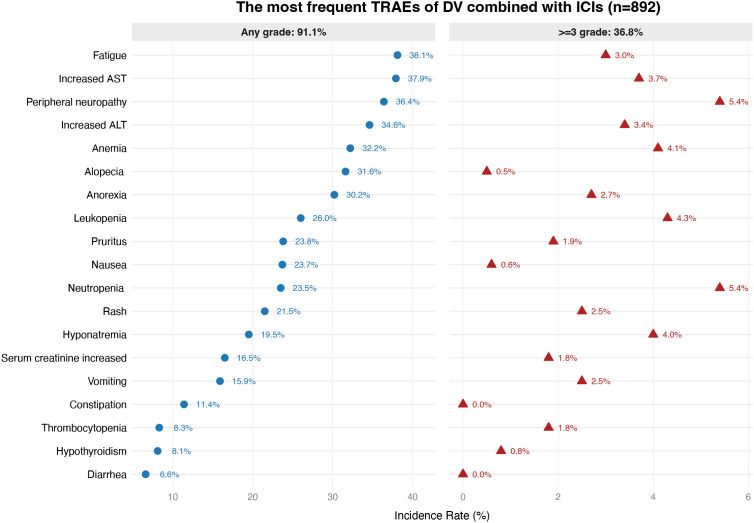
The most frequent treatment-related adverse events of disitamab vedotin combined with immunotherapy in patients with locally advanced or metastatic solid tumors.

Overall, 91.1% of patients receiving DV combined with immunotherapy experienced any grade of AEs, while grade ≥3 AEs occurred in 36.8% of patients. As detailed in **Figure 2**, the most frequent any-grade AEs were fatigue (38.1%), increased aspartate ​aminotransferase (37.9%), peripheral neuropathy (36.4%), and increased alanine ​aminotransferase (34.6%). The most common grade ≥3 AEs included neutropenia (5.4%), peripheral neuropathy (5.4%), and leukopenia (4.3%). Overall, the AE profile was dominated by traditional DV-related toxicities, particularly hematological complications, rather than immune-related AEs.

Owing to variations in drug dosing across tumor types, a subgroup analysis of TRAEs was conducted specifically for UC, which demonstrated consistent findings (**Supplementary Figure 4**). In this subgroup, any-grade and grade ≥3 TRAEs were reported in 90.2% and 36.1% of patients, respectively. The most common any-grade AEs were increased aspartate aminotransferase (38.0%), peripheral neuropathy (37.9%), and fatigue (37.3%), while grade ≥3 AEs primarily included peripheral neuropathy (5.4%), hyponatremia (4.1%), and elevated aspartate aminotransferase (3.7%).

### 3.4 Secondary endpoints: DCR

**Figure 3:**
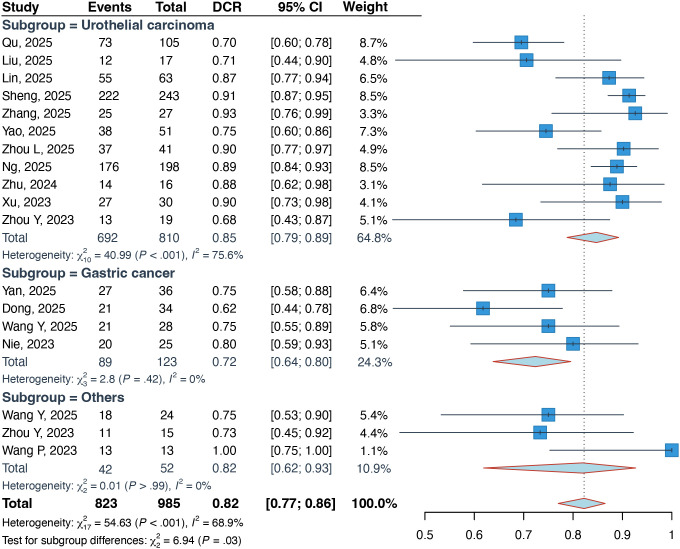
Forest plots about the pooled DCR in patients with locally advanced or metastatic solid tumors. DCR, disease control rate.

Pooled data from 16 studies (n = 985) showed an overall DCR of 82% (95% CI: 77%–86%) (**Figure 3**). Subgroup analysis indicated variations by tumor type, with UC patients achieving a significantly higher DCR of 85% (95% CI: 79%–89%) compared to 72% (95% CI: 64%–80%) in GC patients (P = 0.03).

### 3.5 Secondary endpoints: mPFS

**Figure 4:**
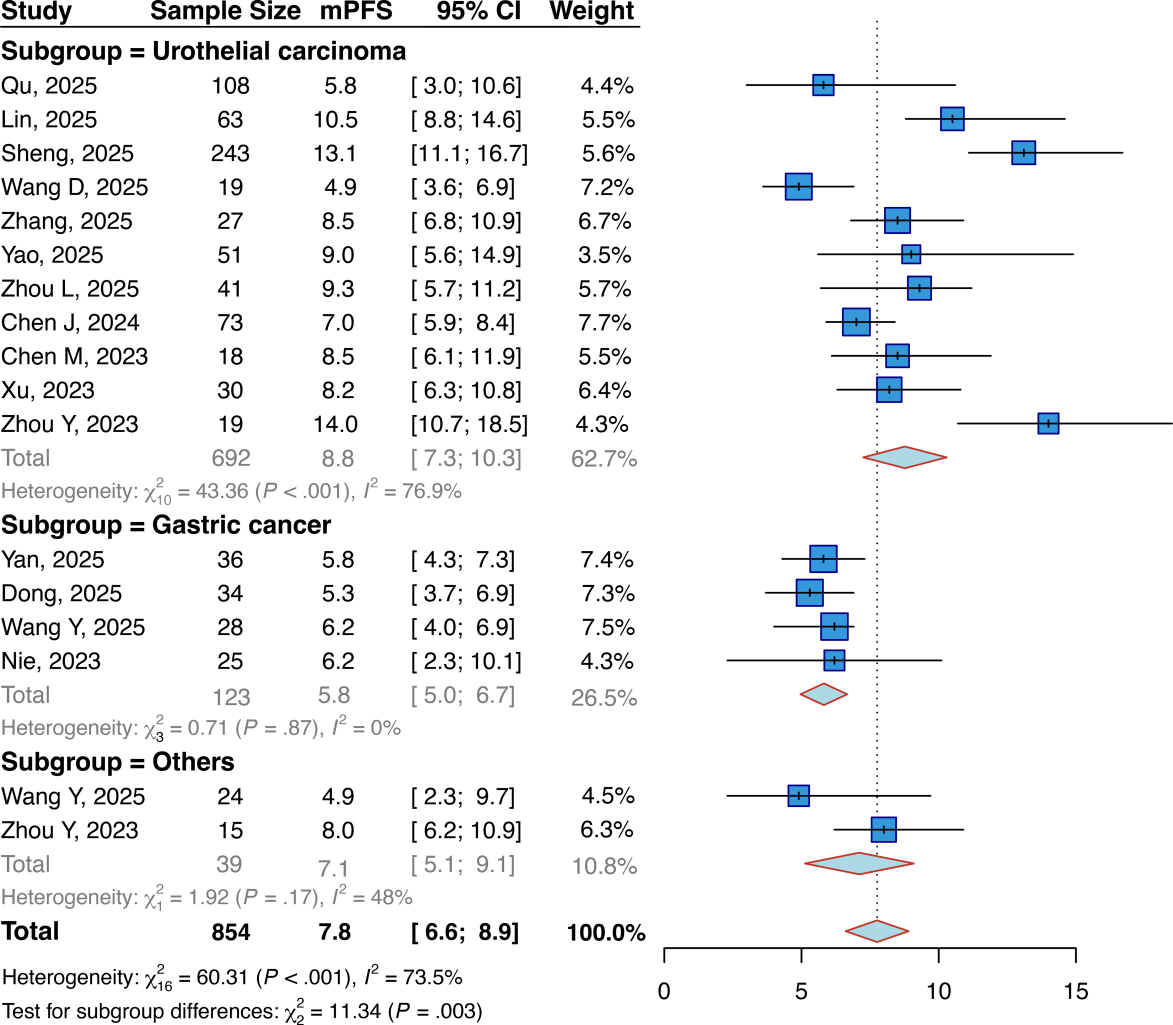
Forest plots about the pooled mPFS in patients with locally advanced or metastatic solid tumors. mPFS, median progression-free survival.

15 studies involving 854 evaluable patients were included in the mPFS analysis. The pooled mPFS was 7.8 months (95% CI: 6.6–8.9) (**Figure 4**). Tumor-based subgroup analysis revealed a significantly longer mPFS in UC patients (8.8 months; 95% CI: 7.3–10.3) than in GC patients (5.8 months; 95% CI: 5.0–6.7; P < 0.01).

### 3.6 Methodological quality and publication bias

The methodological quality of included non-randomized studies was rigorously assessed using the ROBINS-I tool, with detailed results presented in **Supplementary Table 3**. Among the 21 eligible studies, the trial conducted by ​Sheng et al. was a randomized controlled trial and thus not evaluated with ROBINS-I (33). Therefore, the remaining ​20 non-randomized studies​ were systematically assessed.

Of these, ​eight studies​ exhibited a ​serious overall risk of bias (26–28, 30–32, 35, 36), primarily attributable to substantial confounding and selection biases, with occasional concerns in outcome measurement and missing data. ​Nine studies​ demonstrated a ​moderate overall risk, commonly limited by issues in outcome assessment, residual confounding, or deviations from intended interventions (20–25, 29, 34, 38). Only ​three studies​ achieved ​low risk ratings​ across most domains, indicating relatively robust methodological control (14, 15, 37).

The comprehensive assessment of publication bias revealed no significant evidence through either visual inspection of funnel plots (**Supplementary Figure 5**) or formal statistical evaluation using Egger’s linear regression test (**Supplementary Figure 6**; P = 0.19).

## 4 Discussion

This systematic review and meta-analysis represent the first study to evaluated the efficacy and safety of DV in combination with immunotherapy in patients with locally advanced or metastatic solid tumors. Pooled results from 21 studies involving 1183 patients demonstrated an encouraging ORR of 53%, a DCR of 82%, and a mPFS of 7.8 months. The safety profile was manageable, with hematological and neurological events representing the most common treatment-related adverse events. These findings suggest that the combination of DV and ICIs offers a promising therapeutic strategy for this patient population.

Compared to traditional chemotherapy, which typically yields ORRs of 30–50% in advanced solid tumors but is often associated with considerable toxicity (33, 39–41), the DV-ICI regimen not only achieves higher response rates but also exhibits a distinct and manageable safety profile. This superiority is further corroborated by the recent phase III RC48-C016 trial, which directly compared DV plus Toripalimab with platinum-based chemotherapy in previously untreated HER2-expressing locally advanced or metastatic urothelial carcinoma (33). The trial demonstrated a significant improvement in both PFS (median 13.1 vs. 6.5 months; HR 0.36) and overall survival (median 31.5 vs. 16.9 months; HR 0.54) with the combination therapy, establishing a new benchmark for first-line treatment in this setting. In addition, our subgroup analysis observed superior efficacy in the first-line setting (ORR: 71%) compared to later lines (ORR: 52%). Therefore, these results support the consideration of this regimen as a first-line option, particularly in HER2-expressing tumors.

The integration of DV into the therapeutic landscape for certain solid tumors has spurred growing interest in exploring its combination with other treatment modalities to improve clinical outcomes (42–46). Compared with historical results from DV monotherapy in HER2-expressing solid tumors, combining DV with immunotherapy demonstrates significantly enhanced antitumor activity. The synergistic efficacy observed with DV and ICIs is underpinned by multifaceted complementary mechanisms that converge to remodel the tumor immune microenvironment. As a HER2-targeted ADC, DV not only delivers MMAE directly to HER2-expressing tumor cells, inducing caspase-dependent apoptosis and immunogenic cell death, but also elicits broader immunomodulatory effects (13). The ICD triggered by DV involves the release of damage-associated molecular patterns, such as ATP and calreticulin, which act as potent danger signals to recruit and activate dendritic cells. This promotes phagocytosis of tumor antigens and cross-presentation to CD8+ T cells via major histocompatibility complex class I molecules, thereby priming a *de novo* antitumor T-cell response (47). Concurrently, DV’s bystander effect—enabled by the permeable payload MMAE—can eradicate adjacent HER2-low or heterogeneous tumor cells, further amplifying antigen spread and reducing immune escape. In addition, ICIs augment this response by blocking inhibitory checkpoints (e.g., PD-1/PD-L1), which reverses T-cell exhaustion and prevents the engagement of co-inhibitory receptors on activated T cells (48). This dual approach transforms immunologically “cold” tumors into “hot” ones: DV-induced ICD provides the necessary antigenic stimulus and co-stimulatory signals, while ICIs sustain T-cell effector functions by mitigating adaptive resistance. Preclinical models reinforce this synergy; for instance, in HER2-positive murine tumors, DV monotherapy increased tumor-infiltrating lymphocytes and upregulated PD-L1 expression on both tumor and immune cells—a adaptive feedback mechanism that paradoxically enhances susceptibility to anti-PD-1 therapy (49, 50). Furthermore, DV has been shown to activate the cGAS-STING pathway, fostering type I interferon production and natural killer cell recruitment, which synergizes with ICI-mediated T-cell cytotoxicity (51). Despite these compelling mechanisms, robust clinical validation through randomized trials is imperative to confirm the translational relevance of these pathways and optimize combination strategies.

In the safety analysis of this study, it was observed that TRAEs associated with DV combined with ICIs were primarily attributable to DV. Furthermore, during the literature screening process, we noted that in some studies, the incidence of grade ≥3 TRAEs with the combination therapy appeared higher than that reported with DV monotherapy. Although the present analysis did not directly compare TRAE rates between DV-ICI combination and DV monotherapy, the above findings suggest that ICIs may potentially increase the incidence of grade ≥3 TRAEs associated with DV. Possible explanations for this observation include immune system activation by ICIs, which might exacerbate inflammatory responses or alter the tissue tolerance threshold, thereby intensifying the toxic effects of the cytotoxic payload released by DV (48). Additionally, immune-mediated tissue damage may impair the repair mechanisms typically invoked by DV-induced injury, leading to higher-grade adverse events. However, a previous meta-analysis focusing on ADCs combined with immunotherapy in solid tumors demonstrated comparable incidences of grade ≥3 TRAEs between combination therapy and ADC monotherapy (40). It should be noted, however, that this earlier study did not include DV. Therefore, further well-designed studies are warranted to clarify the safety profile of DV in combination with ICIs compared to DV alone (52).

Subgroup analysis revealed that HER2-positive tumors exhibited a significantly higher ORR (67%) compared to HER2-negative cases (50%). This aligns with the known mechanism of action of DV as an HER2-directed antibody-drug conjugate, supporting the dependence on target antigen expression for optimal drug internalization and payload delivery (53). However, the clinically meaningful response observed even in HER2-low populations underscores the potential of DV to address a broader patient group beyond conventional HER2-positive classifications, possibly due to the bystander effect of the membrane-permeable MMAE toxin. Furthermore, the heterogeneity of HER2 expression should also be taken into consideration, as we previously observed in upper tract urothelial carcinoma (54).

The findings from this meta-analysis indicate that the combination of DV and ICIs could represent a new standard of care for certain advanced solid tumors, especially in urothelial carcinoma and HER2-positive gastric cancer. This strategy leverages both targeted cytotoxicity and immunoactivation, potentially transforming outcomes in populations with limited treatment options. Moreover, the tolerable safety profile facilitates clinical applicability and combination feasibility.

Several limitations should be acknowledged. First, the included studies were predominantly retrospective and single-arm, introducing potential selection bias and unmeasured confounding. Second, significant heterogeneity was observed across studies, possibly due to variations in tumor types, HER2 assessment methods, and treatment protocols. Third, the limited number of studies in certain subgroups (e.g., gastric cancer) constrained deeper comparative analyses, and longer-term outcomes such as overall survival could not be robustly evaluated due to insufficient data maturation and inconsistent reporting. Furthermore, the generalizability of our findings may be constrained by the predominance of studies conducted in Chinese populations. Potential influences of genetic background, regional therapeutic practices, and socio-demographic factors on treatment efficacy and safety warrant consideration. Thus, the extrapolation of these results to other ethnic groups requires validation through future multi-regional studies.

## 5 Conclusions​

This study provides a comprehensive synthesis of the current evidence on DV combined with immunotherapy for locally advanced or metastatic solid tumors. The results confirm that this combination regimen yields meaningful clinical efficacy, as evidenced by substantial ORR, DCR, and mPFS, alongside a predictable safety profile primarily characterized by DV-associated toxicities. The enhanced efficacy observed in HER2-positive tumors and in the first-line setting offers valuable guidance for patient selection and clinical trial design. Despite the limitations inherent in the included retrospective studies, the findings strongly suggest that the DV-ICI combination is a viable and promising treatment strategy. Future prospective, randomized trials are warranted to definitively establish its efficacy and safety relative to standard of care, and to further explore predictive biomarkers for response.

## Data Availability

The original contributions presented in the study are included in the article/**Supplementary Material**. Further inquiries can be directed to the corresponding authors.
