# Supplementary material for: The efficacy and safety of disitamab vedotin plus immunotherapy in locally advanced or metastatic solid tumors: a systematic review and meta-analysis
Source: Front Immunol. 2026 Feb 25;17:1763542. doi: 10.3389/fimmu.2026.1763542 (PMC12975876; doi:10.3389/fimmu.2026.1763542)
Supplement: Supplementary file 1 [file DataSheet1.docx]

**Supplementary material**

**The efficacy and safety of disitamab vedotin plus immunotherapy in locally advanced or metastatic solid tumors: a systematic review and meta-analysis**

| Index |
| --- |
| Supplementary Table 1. The PRISMA checklist of the present study. |
| Supplementary Table 2. Summary risk of bias assessment of studies assessed using ROBINS-I tool. |
| Supplementary Fig 1. The specific screening flow chart of included studies. |
| Supplementary Fig 2. Forest plots about the pooled ORR in HER2-positive and HER2-negative patients with locally advanced or metastatic solid tumors. |
| Supplementary Fig 3. Forest plot for the ORR from a pooled analysis of patients with locally advanced or metastatic solid tumors, stratified by treatment line. |
| Supplementary Fig 4. The pooled results about the most frequent treatment-related adverse events of disitamab vedotin combined with immunotherapy in patients with urothelial carcinoma. |
| Supplementary Fig 5. Funnel plots for the included studies. |
| Supplementary Fig 6. Publication bias test of the included studies (p value for egger’ test is 0.17). |

**Supplementary Table 1**. The PRISMA checklist of the present study.

| **Section and Topic** | **Item #** | **Checklist item** | **Location where item is reported** |
| --- | --- | --- | --- |
| **TITLE** | | |  |
| Title | 1 | Identify the report as a systematic review. | Line 1-3 |
| **ABSTRACT** | | |  |
| Abstract | 2 | See the PRISMA 2020 for Abstracts checklist. | Line 4-29 |
| **INTRODUCTION** | | |  |
| Rationale | 3 | Describe the rationale for the review in the context of existing knowledge. | Line 30-59 |
| Objectives | 4 | Provide an explicit statement of the objective(s) or question(s) the review addresses. | Line 60-62 |
| **METHODS** | | |  |
| Eligibility criteria | 5 | Specify the inclusion and exclusion criteria for the review and how studies were grouped for the syntheses. | Line 71-84 |
| Information sources | 6 | Specify all databases, registers, websites, organisations, reference lists and other sources searched or consulted to identify studies. Specify the date when each source was last searched or consulted. | Line 71-74 |
| Search strategy | 7 | Present the full search strategies for all databases, registers and websites, including any filters and limits used. | Line 71-74 |
| Selection process | 8 | Specify the methods used to decide whether a study met the inclusion criteria of the review, including how many reviewers screened each record and each report retrieved, whether they worked independently, and if applicable, details of automation tools used in the process. | Line 73-84 |
| Data collection process | 9 | Specify the methods used to collect data from reports, including how many reviewers collected data from each report, whether they worked independently, any processes for obtaining or confirming data from study investigators, and if applicable, details of automation tools used in the process. | Line 86-94 |
| Data items | 10a | List and define all outcomes for which data were sought. Specify whether all results that were compatible with each outcome domain in each study were sought (e.g. for all measures, time points, analyses), and if not, the methods used to decide which results to collect. | Line 86-94 |
|  | 10b | List and define all other variables for which data were sought (e.g. participant and intervention characteristics, funding sources). Describe any assumptions made about any missing or unclear information. | Line 86-94 |
| Study risk of bias assessment | 11 | Specify the methods used to assess risk of bias in the included studies, including details of the tool(s) used, how many reviewers assessed each study and whether they worked independently, and if applicable, details of automation tools used in the process. | Line 96-103 |
| Effect measures | 12 | Specify for each outcome the effect measure(s) (e.g. risk ratio, mean difference) used in the synthesis or presentation of results. | Line 105-123 |
| Synthesis methods | 13a | Describe the processes used to decide which studies were eligible for each synthesis (e.g. tabulating the study intervention characteristics and comparing against the planned groups for each synthesis (item #5)). | Line 105-123 |
|  | 13b | Describe any methods required to prepare the data for presentation or synthesis, such as handling of missing summary statistics, or data conversions. | Line 105-123 |
|  | 13c | Describe any methods used to tabulate or visually display results of individual studies and syntheses. | Line 105-123 |
|  | 13d | Describe any methods used to synthesize results and provide a rationale for the choice(s). If meta-analysis was performed, describe the model(s), method(s) to identify the presence and extent of statistical heterogeneity, and software package(s) used. | Line 105-123 |
|  | 13e | Describe any methods used to explore possible causes of heterogeneity among study results (e.g. subgroup analysis, meta-regression). | Line 105-123 |
|  | 13f | Describe any sensitivity analyses conducted to assess robustness of the synthesized results. | Line 105-123 |
| Reporting bias assessment | 14 | Describe any methods used to assess risk of bias due to missing results in a synthesis (arising from reporting biases). | Line 105-123 |
| Certainty assessment | 15 | Describe any methods used to assess certainty (or confidence) in the body of evidence for an outcome. | Line 105-123 |
| **RESULTS** | | |  |
| Study selection | 16a | Describe the results of the search and selection process, from the number of records identified in the search to the number of studies included in the review, ideally using a flow diagram. | Line 126-139 |
|  | 16b | Cite studies that might appear to meet the inclusion criteria, but which were excluded, and explain why they were excluded. | Line 126-139 |
| Study characteristics | 17 | Cite each included study and present its characteristics. | Line 126-139 |
| Risk of bias in studies | 18 | Present assessments of risk of bias for each included study. | Line 179-188 |
| Results of individual studies | 19 | For all outcomes, present, for each study: (a) summary statistics for each group (where appropriate) and (b) an effect estimate and its precision (e.g. confidence/credible interval), ideally using structured tables or plots. | Line 126-139 |
| Results of syntheses | 20a | For each synthesis, briefly summarise the characteristics and risk of bias among contributing studies. | Line 141-177 |
|  | 20b | Present results of all statistical syntheses conducted. If meta-analysis was done, present for each the summary estimate and its precision (e.g. confidence/credible interval) and measures of statistical heterogeneity. If comparing groups, describe the direction of the effect. | Line 141-177 |
|  | 20c | Present results of all investigations of possible causes of heterogeneity among study results. | Line 141-177 |
|  | 20d | Present results of all sensitivity analyses conducted to assess the robustness of the synthesized results. | Line 141-177 |
| Reporting biases | 21 | Present assessments of risk of bias due to missing results (arising from reporting biases) for each synthesis assessed. | Line 179-188 |
| Certainty of evidence | 22 | Present assessments of certainty (or confidence) in the body of evidence for each outcome assessed. | Line 179-188 |
| **DISCUSSION** | | |  |
| Discussion | 23a | Provide a general interpretation of the results in the context of other evidence. | Line 190-197 |
|  | 23b | Discuss any limitations of the evidence included in the review. | Line 254-261 |
|  | 23c | Discuss any limitations of the review processes used. | Line 254-261 |
|  | 23d | Discuss implications of the results for practice, policy, and future research. | Line 198--253 |
| **OTHER INFORMATION** | | |  |
| Registration and protocol | 24a | Provide registration information for the review, including register name and registration number, or state that the review was not registered. | Line 64-69 |
|  | 24b | Indicate where the review protocol can be accessed, or state that a protocol was not prepared. | Line 64-69 |
|  | 24c | Describe and explain any amendments to information provided at registration or in the protocol. | Line 64-69 |
| Support | 25 | Describe sources of financial or non-financial support for the review, and the role of the funders or sponsors in the review. | Line 276-278 |
| Competing interests | 26 | Declare any competing interests of review authors. | Line 280-284 |
| Availability of data, code and other materials | 27 | Report which of the following are publicly available and where they can be found: template data collection forms; data extracted from included studies; data used for all analyses; analytic code; any other materials used in the review. | Line 286 |

*From:*  Page MJ, McKenzie JE, Bossuyt PM, Boutron I, Hoffmann TC, Mulrow CD, et al. The PRISMA 2020 statement: an updated guideline for reporting systematic reviews. BMJ 2021;372:n71. doi: 10.1136/bmj.n71

**Supplementary Table 2. Summary risk of bias assessment of studies assessed using ROBINS-I tool.**

| **Author; Year** | **Bias due to  confounding** | **Bias due to selection of participants** | **Bias in classification  of interventions** | **Bias due to deviations from intended interventions** | **Bias due to  missing data** | **Bias in measurement  of outcomes** | **Bias in selection of  the reported result** | **Overall risk of bias** |
| --- | --- | --- | --- | --- | --- | --- | --- | --- |
| Lin, 2025 | Moderate | Moderate | Low | Moderate | Moderate | Moderate | Moderate | Moderate |
| Qu, 2025 | Low | Low | Low | Moderate | Low | Low | Moderate | Low |
| Liu, 2025 | Serious | Serious | Moderate | Serious | Serious | Serious | Serious | Serious |
| Zhou T, 2025 | Serious | Moderate | Low | Moderate | Serious | Serious | Moderate | Serious |
| Yan, 2025 | Moderate | Moderate | Moderate | Moderate | Moderate | Moderate | Moderate | Moderate |
| Wang D, 2025 | Moderate | Moderate | Low | Low | Moderate | Moderate | Low | Moderate |
| Yao, 2025 | Moderate | Moderate | Low | Low | Moderate | Low | Low | Moderate |
| Zhang, 2025 | Moderate | Moderate | Low | Low | Moderate | Low | Low | Moderate |
| Zhou L, 2025 | Low | Low | Low | Low | Low | Moderate | Low | Low |
| Ng, 2025 | Moderate | Moderate | Low | Low | Moderate | Low | Low | Moderate |
| Ge, 2025 | Moderate | Moderate | Low | Low | Low | Moderate | Low | Moderate |
| Dong, 2025 | Moderate | Moderate | Low | Moderate | Moderate | Low | Low | Moderate |
| Wang Y, 2025 | Low | Low | Low | Low | Low | Low | Moderate | Low |
| Chen J, 2024 | Serious | Moderate | Low | Moderate | Moderate | Low | Moderate | Serious |
| Zhu, 2024 | Moderate | Moderate | Low | Low | Low | Moderate | Low | Moderate |
| Chen M, 2023 | Serious | Moderate | Low | Moderate | Low | Moderate | Low | Serious |
| Xu; 2023 | Serious | Moderate | Low | Moderate | Low | Moderate | Low | Serious |
| Zhou Y, 2023 | Moderate | Serious | Low | Low | Serious | Low | Serious | Serious |
| Wang P, 2023 | Serious | Moderate | Low | Low | Low | Serious | Low | Serious |
| Nie, 2023 | Serious | Moderate | Low | Low | Low | Serious | Low | Serious |

**Supplementary Fig 1.** The specific screening flow chart of included studies.

**Supplementary Fig 2.** Forest plots about the pooled ORR in HER2-positive and HER2-negative patients with locally advanced or metastatic solid tumors.

ORR = objective response rate.

**Supplementary Fig 3.** Forest plots for the ORR from a pooled analysis of patients with locally advanced or metastatic solid tumors, stratified by treatment line.

ORR = objective response rate.

**Supplementary Fig 4.** The pooled results about the most frequent treatment-related adverse events of disitamab vedotin combined with immunotherapy in patients with urothelial carcinoma.

**Supplementary Fig 5.** Funnel plots for the included studies.

**Supplementary Fig 6.** Publication bias test of the included studies (p value for egger’ test is 0.19).
